# Supplementary material for: Advanced Removal of Dyes with Tuning Carbon/TiO2 Composite Properties
Source: Nanomaterials (Basel). 2024 Feb 3;14(3):309. doi: 10.3390/nano14030309 (PMC10856939; doi:10.3390/nano14030309)
Supplement: Supplementary file 1 [file nanomaterials-14-00309-s001.zip › nanomaterials-2804311-supplementary.pdf]

Supplementary materials

Advanced Removal of Dyes with Tuning Carbon/TiO_2 Composite Properties

Halyna Bodnar Yankovych [1,*], Coset Abreu-Jaureguí [2], Judit Farrando-Perez [2], Inna Melnyk [1], Miroslava Václavíková [1] and Joaquín Silvestre-Albero [2,*]

[1] Institute of Geotechnics, Slovak Academy of Sciences, Watsonova 45, 04001 Košice, Slovakia; in.melnyk@gmail.com (I.M.); vaclavik@saske.sk (M.V.)

[2] Laboratorio de Materiales Avanzados, Departamento de Química Inorgánica-Instituto Universitario de Materiales, Universidad de Alicante, E-03690 San Vicente del Raspeig, Spain; coset.abreu@ua.es (C.A.-J.); judit.farrando@ua.es (J.F.-P.)

* Correspondence: halynayankovych@gmail.com (H.B.Y.); joaquin.silvestre@ua.es (J.S.-A.)

Table S1. Physico-chemical properties of dyes used (according to ChemSpider Dataset)

Compound name	Physico-chemical properties	Structural formula
Acid Red 88<br>IUPAC [sodium;4-[(2-hydroxynaphthalen-1-yl)diazenyl]naphthalene-1-sulfonate]	$C_{20}H_{13}N_2NaO_4S$<br>M=400.4 g·mol^{-1}<br>λ_{max}=505 nm<br>pK$_a$=10.7<br>**anionic dye**	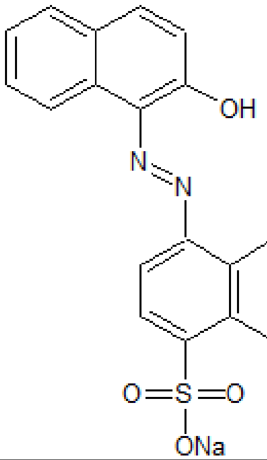
Basic Red 13<br>IUPAC<br>[2-(2-(4-((2-Chloroethyl) methylamino)phenyl) vinyl)-1,3,3-trimethyl-3H-indolium chloride]	$C_{22}H_{26}Cl_2N_2$<br>M=389.4 g·mol^{-1}<br>λ_{max}=523 nm<br>pK$_a$=2.9<br>**cationic dye**	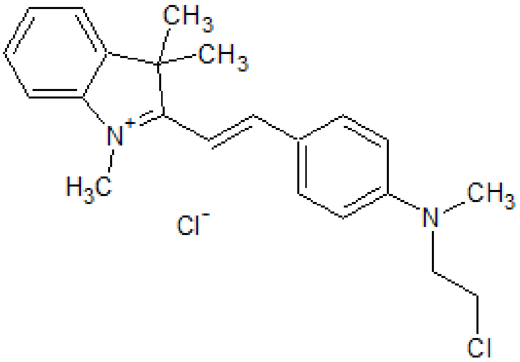
Basic Red 5<br>IUPAC [3-Amino-7-dimethylamino-2-methyl phenazine hydrochloride]	$C_{15}H_{17}N_4 \cdot HCl$<br>M=288.8 g·mol^{-1}<br>$\lambda_{max}$=537 nm<br>pK$_a$=6.7, 7.4<br>**neutral dye**	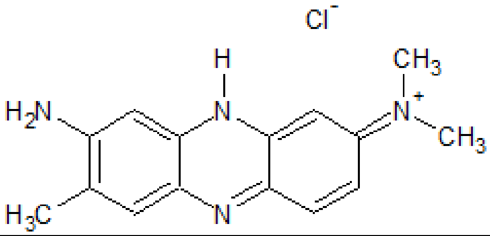

Table S2. EDS analysis of GAC

Element	Mass%	Atom%
C	71.74±0.08	79.44±0.09
O	20.00±0.12	16.63±0.10
Al	3.86±0.02	1.90±0.01
Si	3.79±0.02	1.79±0.01
S	0.33±0.01	0.13±0.00
Ca	0.30±0.01	0.10±0.00
Total	100.00	100.00

Table S3. EDS analysis of carbon_TiO_2

Element	Mass%	Atom%
C	31.49±0.05	45.36±0.07
O	40.57±0.15	43.88±0.17
Al	0.98±0.01	0.63±0.01
Si	1.09±0.01	0.67±0.01
P	0.18±0.01	0.10±0.00
S	0.72±0.01	0.39±0.00
Ti	24.20±0.06	8.74±0.02
Fe	0.76±0.02	0.24±0.01
Total	100.00	100.00

Table S4. EDS analysis of carbon_TiO_2_Zr

Element	Mass%	Atom%
C	23.51±0.04	36.65±0.06
O	40.89±0.13	47.84±0.15
Mg	0.22±0.01	0.17±0.01
Al	1.42±0.01	0.99±0.01
Si	1.97±0.01	1.31±0.01
S	3.02±0.01	1.77±0.01
Ca	4.32±0.02	2.02±0.01
Ti	22.00±0.05	8.60±0.02
Fe	0.82±0.02	0.27±0.01
Zr	1.82±0.02	0.37±0.00
Total	100.00	100.00

Table S5. EDS analysis of carbon_TiO_2_Ce

Element	Mass%	Atom%
C	25.93±0.04	38.88±0.06
O	42.89±0.13	48.29±0.14
Al	1.36±0.01	0.91±0.01
Si	2.01±0.01	1.29±0.01
S	0.88±0.01	0.49±0.00
Ca	0.71±0.01	0.32±0.00
Ti	25.52±0.05	9.60±0.02
Fe	0.69±0.01	0.22±0.00
Total	100.00	100.00

Table S6. EDS analysis of carbon_TiO_2_Cu

Element	Mass%	Atom%
C	28.98±0.04	42.55±0.06
O	41.43±0.13	45.67±0.15
Al	1.18±0.01	0.77±0.01
Si	1.70±0.01	1.07±0.01
S	0.56±0.01	0.31±0.00
Ti	26.14±0.05	9.63±0.02
Total	100.00	100.00

Table S7. XPS summary of elements content in samples, at%

% Atomic	C	OX	Si	N	S	Ti	Al	Ce	Zr	Cu
GAC	95.0	4.2	0.3	0	0.2	-	0.3	-	-	-
carbon_TiO_2	49.3	35.8	2.1	0.8	0.7	9.9	1.5	-	-	-
carbon_TiO_2_Zr	73.7	20.5	0.2	0.9	0.3	4.0	0	-	0.3	-
carbon_TiO_2_Ce	72.1	21.1	1.2	0.7	0.3	3.5	1.1	0	-	-
carbon_TiO_2_Cu	69.3	25.4	0.4	1.4	0.2	2.8	0.6	-	-	0

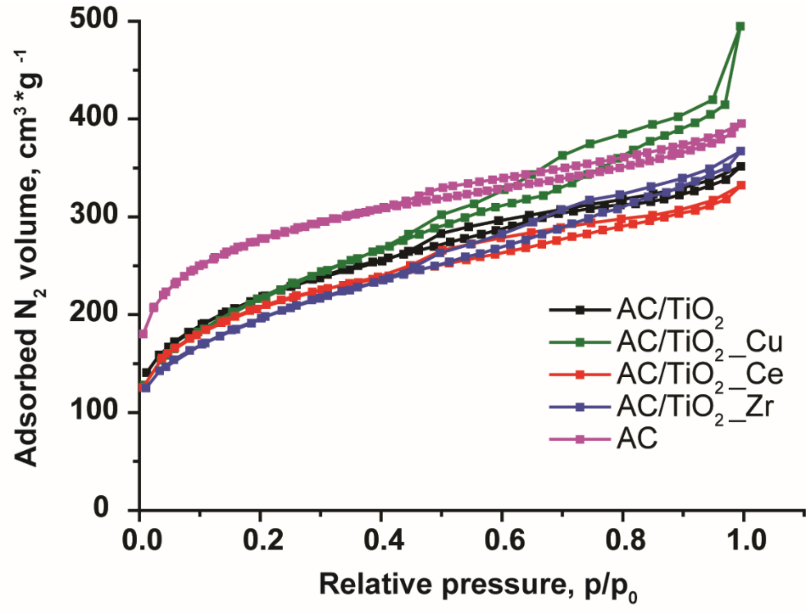

Figure S1. N_2 adsorption-desorption isotherms of used materials
